# Schwannoma: A Rare Cause of Perineal Pain

**DOI:** 10.5334/jbsr.2738

**Published:** 2022-02-18

**Authors:** Ophélye Chiabai, Etienne Marbaix, Cristina Dragean

**Affiliations:** 1 UCL, BE

**Keywords:** schwannoma, pelvic, rectovaginal septum

## Abstract

**Teaching point:** Unexplained persistent perineal pain poses a differential diagnosis, including pelvic nerve lesions. The rare occurrence of pelvic schwannoma is easily shown by a MRI as a T2-hyperintense enhancing mass.

## Case History

A 60-year-old woman in good general condition complained of right-sided perineal pain in sitting position. The clinical examination found an ovoid mass to the right of the rectovaginal septum, palpation of which triggered very painful dysesthesia.

**Figure 1:**
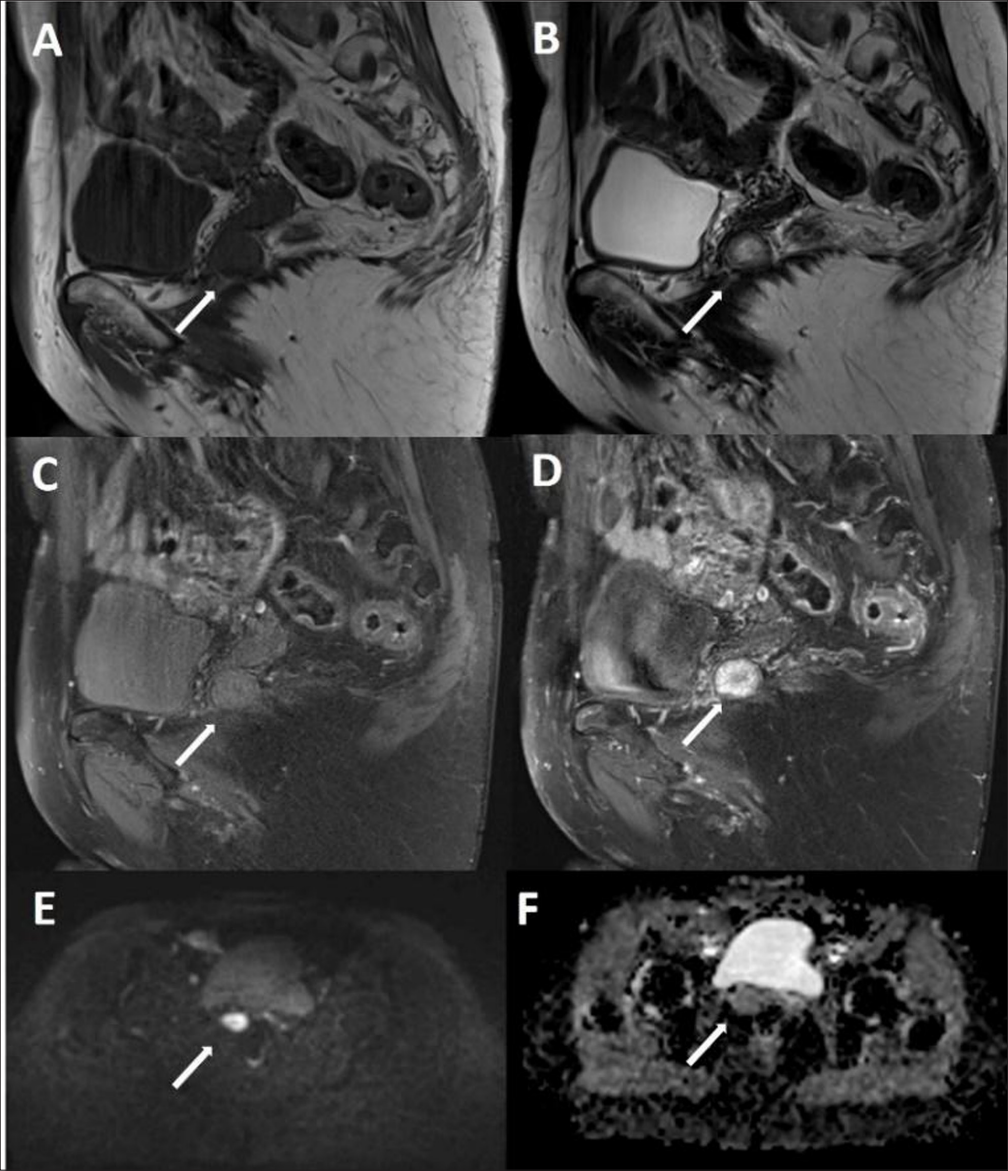


Subsequent magnetic resonance imaging (MRI) (***Figure 1***) in the sagittal (A–D) and axial (E, F) planes showed a well-defined 20 mm mass on the right postero-lateral side of the rectovaginal septum, making a protrusion in the pouch of Douglas. This anomaly is T1 isointense (***Figure 1A*** and ***C***); T2 hyperintense, with a well delimited hypointense periphery (***Figure 1B***); causes restriction on diffusion-weighted imaging (***Figure 1E***), without apparent diffusion coefficient decrease (***Figure 1F***); and lastly, intensely enhances after injection of gadolinium (***Figure 1D***).

These findings were consistent with the preoperative diagnosis of schwannoma.

The mass was completely excised and the symptoms disappeared after the surgery. Histopathology confirmed the diagnosis of schwannoma by displaying an oval-shaped lesion contained in a capsule (epineurium) and eccentric to the nerve.

## Commentary

Perineal pain is a frequent complaint especially amongst women, and it can have different origins: ligament, muscular, nervous, hernia, prolapse, infectious, inflammatory or tumoral.

In the case of a perineal mass, the most frequent benign nodule is endometriosis which typically has, unlike in the present case, a high T1 signal and/or a low T2 signal depending on its subtype.

Schwannomas, though rare in the perineal region, are the most common benign tumors of the peripheral nerves, recognizable on MRI, as in the present case, with intensely high T2 signal and enhancement after injection of contrast [1].

Malignant masses such as adenocarcinoma, GIST, and metastasis have less-intense T2 signal and heterogenous enhancement. Furthermore, other imaging findings (lesion contour, adjacent organ invasion, lymph nodes, etc.) and patient history are suggestive.
